# StCDPK3 Phosphorylates *In Vitro* Two Transcription Factors Involved in GA and ABA Signaling in Potato: StRSG1 and StABF1

**DOI:** 10.1371/journal.pone.0167389

**Published:** 2016-12-01

**Authors:** Carolina Grandellis, Elisa Fantino, María Noelia Muñiz García, Magalí Graciela Bialer, Franco Santin, Daniela Andrea Capiati, Rita María Ulloa

**Affiliations:** 1 Institute of Genetic Engineering and Molecular Biology (INGEBI), National Research Council (CONICET) Vuelta de Obligado, 2^do^ piso, Buenos Aires, Argentina; 2 Biochemistry Department, School of Exact and Natural Sciences, University of Buenos Aires, Buenos Aires, Argentina; National Taiwan University, TAIWAN

## Abstract

Calcium-dependent protein kinases, CDPKs, decode calcium (Ca^2+^) transients and initiate downstream responses in plants. In order to understand how CDPKs affect plant physiology, their specific target proteins must be identified. In tobacco, the bZIP transcription factor Repression of Shoot Growth (NtRSG) that modulates gibberellin (GA) content is a specific target of NtCDPK1. StCDPK3 from potato is homologous (88% identical) to NtCDPK1 even in its N-terminal variable domain. In this work, we observe that NtRSG is also phosphorylated by StCDPK3. The potato RSG family of transcription factors is composed of three members that share similar features. The closest homologue to *NtRSG*, which was named *StRSG1*, was amplified and sequenced. qRT-PCR data indicate that *StRSG1* is mainly expressed in petioles, stems, lateral buds, and roots. In addition, GA treatment affected *StRSG1* expression. *StCDPK3* transcripts were detected in leaves, petioles, stolons, roots, and dormant tubers, and transcript levels were modified in response to GA. The recombinant StRSG1-GST protein was produced and tested as a substrate for StCDPK3 and StCDPK1. 6xHisStCDPK3 was able to phosphorylate the potato StRSG1 in a Ca^2+^-dependent way, while 6xHisStCDPK1 could not. StCDPK3 also interacts and phosphorylates the transcription factor StABF1 (ABRE binding factor 1) involved in ABA signaling, as shown by EMSA and phosphorylation assays. *StABF1* transcripts were mainly detected in roots, stems, and stolons. Our data suggest that StCDPK3 could be involved in the cross-talk between ABA and GA signaling at the onset of tuber development.

## Introduction

Plant signaling involves the interaction of several components and second messengers such as calcium (Ca^2+^). The multigene family of calcium-dependent protein kinases (CDPKs) encodes Ca^2+^ sensor/protein kinase effectors, that are ideal candidates for perceiving intracellular changes in Ca^2+^ concentration and translating them into specific phosphorylation events [1]. Substrate identification represents a critical task for understanding any protein kinase-based signal transduction pathway. CDPKs exhibit overlapping and distinct expression patterns, subcellular localizations, substrate specificities, and Ca^2+^ sensitivities. These kinases regulate the activity, localization, and lifetime of enzymes, channels, and transcription factors (TFs) by phosphorylating specific serine and threonine residues on their target sequences [2,3]. The first level of substrate specificity arises from the interaction between the active site of the kinase and the amino acid sequences surrounding the phosphorylation site of the substrate [4]. Since the catalytic domains of CDPK isoforms are highly conserved, it seems unlikely that CDPKs would have distinguishable substrate specificities. However, CDPK isoforms from different species were reported to play distinct physiological functions [5–12]. As an example, four CPKs from *Arabidopsis* have significant differences in substrate specificity [13]. In addition, the N-terminal variable (NTV) domain of NtCDPK1 was shown to play an essential role in the specific recognition of the substrate [14].

Several TFs are CDPK targets and often become phosphorylated in the plant cell thus activating or inactivating downstream responses [15–17]. TFs are classified according to their DNA binding domains. In particular, basic region/leucine zipper (bZIP) TFs have a region that binds DNA and a leucine zipper dimerization motif. In plants, bZIP TFs regulate several processes including pathogen defense, light and stress signaling, seed maturation, and flower development [18]. The tobacco bZIP TF *Nicotiana tabacum* Repression of Shoot Growth (NtRSG) regulates the morphology of plants by controlling the endogenous amounts of gibberellins (GAs) [19]. NtRSG binds to the *NtGA20ox1* promoter *in vivo* in response to a decrease in GA levels and this binding is abolished within 3 h after GA treatment [20]. Besides, NtRSG is negatively regulated by 14-3-3 signaling proteins [21]. The 14-3-3 proteins emerged as phosphorylation-dependent regulators of hormone and light signaling in plants [22–24]. The interaction between 14-3-3s and target proteins generally occurs at a conserved 14-3-3 binding motif (RSXpSXP and RXY/FXpSXP) where pT and pS denote phosphorylated threonine or serine residues. The 14-3-3 protein binds to NtRSG depending on the phosphorylation status of Ser-114, and sequesters NtRSG in the cytoplasm so that it is unable to regulate its target genes [21,25]. NtCDPK1 interacts with NtRSG both *in vivo* and *in vitro*, and specifically phosphorylates Ser-114 of NtRSG *in vitro* [16].

CDPKs have also been implicated in abscisic acid (ABA) signaling, and overexpression of CDPKs has been shown to activate ABA-regulated promoters [26]. The bZip TFs ABRE-binding factors (ABFs) bind to ABA-responsive elements (ABRE), present in the promoters of ABA-responsive genes, and upregulate their transcription [27]. In *Arabidopsis thaliana*, ABA induced the activation of two homologous CDPKs (CPK4 and CPK11) that phosphorylate ABF1 and ABF4 *in vitro*. Double mutants of the two *CDPK* genes had stronger ABA- and salt-responsive phenotypes than the single mutants suggesting that these kinases may regulate ABA signaling through these TFs [6].

ABA and GAs antagonistically mediate numerous physiological processes, and their optimal balance is essential for normal plant development. In potato, GA is a dominant negative regulator that promotes stolon elongation and inhibits tuber formation [28–31]. In contrast, ABA acts as a tuberization-promoting factor; its application accelerates tuberization in some potato varieties [30,32]. ABA content and ABA/GA ratio increased, and stolon apical growth was retarded, in *andigena* potato leaves grown under tuber-inducing conditions [33]. The function of ABA in tuber development is not clear, and it has been proposed that it promotes tuberization by counteracting the inhibitory effect of GAs [30,34]. Antagonism often involves crosstalk points, where two signaling pathways are interconnected. However, the molecular mechanisms underlying ABA/GA antagonism are not completely elucidated. In Arabidopsis, the AP2/ERF transcription factor ABA-INSENSITIVE 4 (ABI4) is a central factor in GA/ABA homeostasis [35,36]. Also, reactive oxygen species and ascorbic acid were reported to mediate this antagonism during seed germination in rice [37].

The potato CDPK isoforms *Solanum tuberosum* St*CDPK*1, 2, and 3 are differentially expressed in tuberizing stolons [38–43]. These three isoforms are closely related, and belong to group IIa of CDPKs [44]; however, they differ in their kinetic parameters. StCDPK3 recombinant protein (6xHisStCDPK3) displays higher affinity for ATP, and while its kinase activity is Ca^2+^-dependent, autophosphorylation is Ca^2+^ independent [43]. St*CDPK*3 promoter∷GUS transgenic potato lines indicated that St*CDPK*3 is expressed in actively growing organs; St*CDPK*3 expression was positively modulated by ABA (ABREs are present in its promoter) while a reduction in *StCDPK3* transcripts was observed within 6h of GA application to *in vitro* grown potato plants [43]. In this report, we show that 6xHisStCDPK3 phosphorylates the potato bZIP TFs StRSG1 and StABF1 in a Ca^2+^-dependent manner suggesting that StCDPK3 could be an important point of cross-talk between the ABA and GA signaling pathways mediated by both TFs.

## Results

### RSG family and genomic context in potato

In order to identify the RSG TF family in potato, the tobacco NtRSG coding sequence was blasted against the *Solanum phureja* database (http://potato.plantbiology.msu.edu/cgi-bin/gbrowse/potato/). Specific oligonucleotides were designed to amplify the complete coding sequence of the potato homolog using a *S*. *tuberosum* cDNA library as template. The open reading frame (1014 bp) encodes a slightly acidic (pI = 6.46) protein of 340 amino acids with a basic leucine zipper (bZIP) conserved domain (positions 182 to 245) that was designated StRSG1 (GenBank HQ335343.1).

StRSG1 protein sequence was blasted (BLASTP 2.2.26) against the PGSC DM v3.4 pep.fasta database (http://solanaceae.plantbiology.msu.edu/pgsc_download.shtml). As observed, PGSC0003DMP400001527 is 100% identical to StRSG1, while three other bZIP transcriptional activator RSG proteins also present high identity. In addition, other six TFs (two RF2a, two RF2b, one VSF-1, and a DNA binding protein) share 56 to 47% identity with StRSG1 (Q coverage: 27 to 92%). When the blast search was conducted using only the bZIP domain as query, Q coverage was 100%, and identity increased from 50 to 78% (S1 Table).

**Fig 1:**
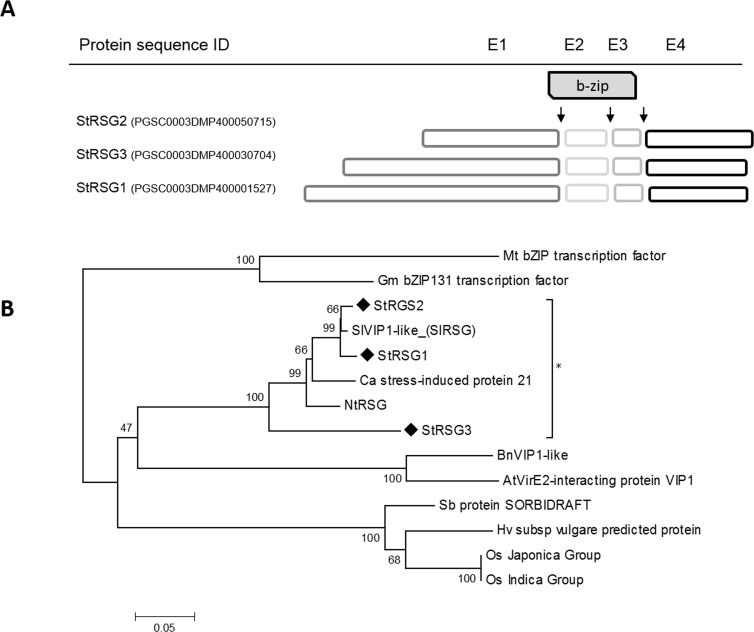
Structure and phylogenetic analysis of RSG proteins. (A) Schematic model of StRSG proteins indicating the region spanned by each exon (E1-4: exons 1–4.); arrows indicate intron positions. The grey box indicates the bZIP domain. Exons lengths are scaled. Intron lengths are indicated in S2 Table. (B) Evolutionary relationships of RSGs full amino acid sequences from *S*. *tuberosum* StRSG1 (HQ335343.1; PGSC0003DMP400001527), StRSG2 (PGSC0003DMP400050715), and StRSG3 (PGSC0003DMP400030704), *Solanum lycopersicum* SlVIP1-like (XP_004237777.1), *Nicotiana tabacum* NtRSG (BAA97100.1), *Capsicum annuum* stress-induced protein 21 (AHI85726.1), *Arabidopsis thaliana* VirE2-interacting protein VIP1 (AAM63070.1), *Brassica napus* TF VIP1-like (NP_001303096.1), *Glycine max* bZIP TF bZIP131 (NP_001237194.1), *Medicago truncatula* bZIP (XP_013451804.1), *Oryza sativa* Os_12g0162500 Japonica Group, a hypothetical protein (OsI_37568) belonging to Indica Group, *Hordeum vulgare* predicted protein (BAK03813.1), and *Sorghum bicolor* hypothetical protein SORBIDRAFT (XP_002441871.1). Amino acid sequences were aligned using ClustalW. The evolutionary history was inferred using the Neighbor-Joining method. The tree was constructed after a bootstrap test (n = 500), the percentage of replicate trees are shown next to the branches. The evolutionary distances were computed using the JTT matrix-based method and are in the units of the number of amino acid substitutions per site. Evolutionary analyses were conducted with MEGA6.

*StRSG1* transcripts share 99% identity with PGSC0003DMT400002100 (identities = 1013/1014, gaps = 1/1014) corresponding to gene PGSC0003DMG400000799 (*StRSG1*) that is localized in chromosome 4 at position 59170 kbp and is 6300 bp long (S2 Table). By overlapping the coding sequence with the genomic scaffold, we identified four exons and three introns, as well as the common and conserved splicing sites. Exons 1, 2, and 3 compose the conserved bZIP domain (64 amino acids, Fig 1A). Only 1.5 kbp upstream *StRSG1*, there is a putative *cytochrome P450* (CYP450) gene sequence (PGSC0003DMG400000798) in the same orientation, and further upstream a putative *serine decarboxylase* (*SDC*) gene sequence (PGSC0003GMG400000785) in tail to tail (TT) orientation. While, 13.6 kbp downstream, there is a putative gene sequence encoding a U3 small nucleolar RNA-associated protein, in the same orientation as *StRSG1* (S1A Fig).

As mentioned, two other gene sequences encoding RSG transcriptional activators were identified (S2 Table). *StRSG2* also maps in chromosome 4 (position 59453 kbp) and is very similar (Identities 95%, Q coverage 93%) to *StRSG1*. According to the PGSC, two transcripts can be generated from *StRSG2;* one is predicted to encode a basic protein (pI = 9.47) of 261 amino acid (StRSG2), and the other one is slightly shorter and lacks the fourth exon (S2 Table). Moreover, 1.6 kbp downstream to *StRSG2*, there are two putative gene sequences (PGSC003DMG400041269 and PGSC003DMG400029122) encoding a CYP450, and a SDC respectively. *CYP450* is in the same orientation as *StRSG2*, while *SDC* is in a TT orientation. Additionally, several actin genes are located 20 kbp upstream (S1B Fig). Alignment of the 100 kb sequences surrounding *StRSG1* and *StRSG2* using blast2seq (http://blast.ncbi.nlm.nih.gov) show that an inverted duplication (96% identity) of a 31.2 kb region encompassing *RSG*, *CYP450*, and *SDC* genes occurred in chromosome 4. These inverted repeats are separated by a 273 kbp spacer region (S1 Fig, upper panel). On the other hand, *StRSG3* gene, localized on chromosome 6, encodes another RSG bZIP protein of 326 amino acids (pI = 7.15) (S2 Table). The three *RSG* genes and the genes encoding RF2a, RF2b, and VSF-1 TFs present the four exons/three introns organization of group I of bZIP TFs [45] and share common intron positions (Fig 1A, S2 Table).

The potato RSGs were compared with those from other monocots and dicots. As observed in the phylogenetic tree generated from the alignments of the full-length protein sequences (Fig 1B), the potato RSGs cluster with those from other solanaceae such as tobacco, tomato, and pepper. StRSG1 and the predicted TF VIP1-like from tomato, SlRSG, only differ in eight amino acids. On the other hand, RSGs from *A*. *thaliana* and *Brassica napus* cluster together, as well as RSGs from *Glycine max* and *Medicago truncatula*. Moreover, RSG from monocots (sorghum, barley, and rice) are clustered in a different group. This suggests that RSGs are strongly conserved among species from the same family and to a lesser extent in higher groups as dicots and monocots.

### StCDPK3 phosphorylates NtRSG and StRSG *in vitro*

StRSG1 and NtRSG TFs share 86% protein identity and 89% similarity, and both contain a conserved serine residue at positions 114 (NtRSG) or 107 (StRSG1) in the context P**L**NHF**R**SL**S**VDA predicted to be a phosphorylation site for CDPKs **(L**X**H**X**R**XX**S**/**T****)**. Phosphorylation of Ser-114 by NtCDPK1 promotes 14-3-3 binding to NtRSG [46]. The other members of the potato RSG family also contain the conserved serine at positions 30 for StRSG2 or 104 for StRSG3 in the same context. In addition, NetPhos.2 [47] predicted 19 serines, 8 threonines and 1 tyrosine phosphorylation sites in StRSG1 sequence. Five serine residues (Ser-39, Ser-71, Ser-141, Ser-159 and Ser-236) had higher scores than 0.9 while among threonines, Thr-125 had the highest score (0.798). However, NetPhos.2 did not detect Ser-107 as a probable phosphorylation site.

**Fig 2:**
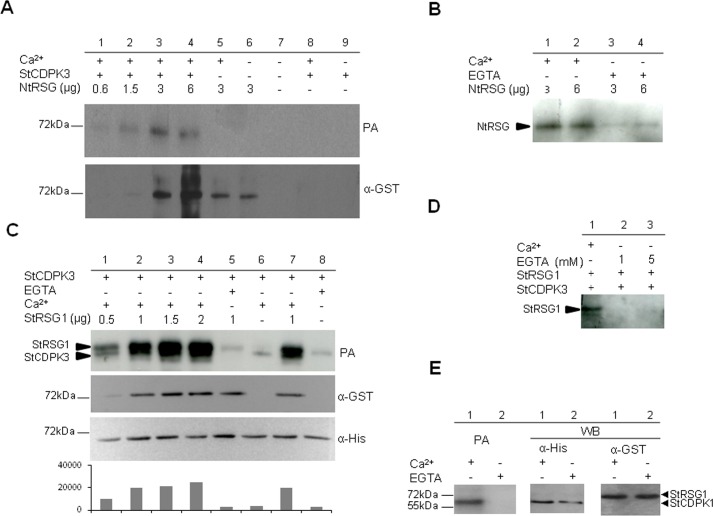
StCDPK3 phosphorylates NtRSG and StRSG1 *in vitro*. Phosphorylation assays (PA) with ATPγP^32^ were performed using NtRSG-GST (A, B) or StRSG1-GST (C, D, E) as substrates and 6xHisStCDPK3 (A-D) or 6xHisStCDPK1 (E) as the enzyme source. Samples were analyzed by 10% SDS-PAGE. StCDPK3 kinase activity was previously confirmed using Syntide-2 as a positive control and GST alone as a negative control [43]. A. Upper panel: different amounts of NtRSG (lanes 1 to 6) were incubated with 50 ng of 6xHisStCDPK3 (lanes 1 to 4) in the presence of 20 μM Ca^2+^ (lanes 1 to 5). Autophosphorylation of 6xHisStCDPK3 was performed in the presence or absence of Ca^2+^ (lanes 8 and 9 respectively). Lower panel: western blot revealed with an anti-GST antibody (1:4000, α-GST). B. Phosphorylation of NtSRG by StCDPK3 in the presence of 20 μM Ca^2+^ (lanes 1 and 2) or 1 mM EGTA (lanes 3 and 4). C. Upper panel: different amounts of StRSG1 (lanes 1 to 5 and 7) were incubated with 6xHisStCDPK3 (100 ng) in the presence of 20 μM Ca^2+^ (lanes 1 to 4 and 7) or 1 mM EGTA (lane 5). Additionally StCDPK3 was autophosphorylated in the presence of 20 μM Ca^2+^ (lane 6) or 1 mM EGTA (lane 8). Middle and lower panels: western blots revealed with anti-GST (1:4000) and anti-His (1:5000, α-His) antibodies, respectively. Image J Software was used to estimate band intensities (AU = arbitrary units). D. Phosphorylation of StRSG1 (0.5 μg) by StCDPK3 (50 ng) in the presence of 20 μM Ca^2+^ (lane 1) or 1 and 5 mM EGTA (lanes 2 and 3). E. StRSG1 (2 μg) was incubated with 6xHisStCDPK1 (100 ng) in the presence of 20 μM Ca^2+^ (lane 1) or 1 mM EGTA (lane 2) (left panel). Middle and right panels: western blot with anti-His and anti-GST antibodies, respectively. The same reaction mixture was used in all PAs.

The NTV domain of NtCDPK1 plays an essential role in the specific recognition of NtRSG [46,48]. StCDPK3 is homologous (88% identity) to the tobacco NtCDPK1 isoform, even when comparing their NTV domains (identity 68/109; 62%). Thus, the heterologous NtRSG-GST recombinant protein (MW = 64.22 kDa; apparent MW~72kDa) was evaluated as a phosphate acceptor for 6xHisStCDPK3 [43]. As observed, StCDPK3 was able to phosphorylate NtRSG in the presence of Ca^2+^ (Fig 2A). The phosphorylated bands correspond to NtRSG since no band was observed in its absence (lanes 8 and 9). StCDPK3 undergoes autophosphorylation [43], however, the quantity used in the assay (50 ng) was not enough to detect it. In order to confirm that RSG phosphorylation was Ca^2+^-dependent, increasing amounts of NtRSG were incubated with StCDPK3 in the presence of Ca^2+^ or EGTA. As observed, NtRSG phosphorylation was very low in the presence of EGTA (Fig 2B).

Then, we tested whether 6xHisStCDPK3 was able to phosphorylate the potato StRSG1-GST recombinant protein *in vitro*. Increasing amounts of StRSG1-GST and a constant amount of 6xHisStCDPK3 were used in the phosphorylation assay (Fig 2C, upper panel). Both proteins possess similar molecular weights (estimated MW for 6xHisStCDPK3 is 63 kDa, and for StRSG1-GST is 64.47 kDa), so anti-GST and anti-His antibodies were used to differentiate them (Fig 2C, middle and lower panels). As expected, StRSG1-GST phosphorylation was more intense with increasing amounts of the substrate (Fig 2C, lanes 1–4). StRSG1 was phosphorylated by StCDPK3 when Ca^2+^ was added to the reaction (Fig 2C and Fig 2D, lane 1), but no phosphorylation was detected in the presence of 1 or 5 mM EGTA (Fig 2D, lanes 2 and 3), further confirming the Ca^2+^ dependency. In addition, autophosphorylation of 6xHisStCDPK3 was more intense in the presence of StRSG1 (Fig 2C, lane 7) than in its absence (lanes 6 and 8), suggesting that the substrate could modulate the kinase autophosphorylation status. A similar phosphorylation assay was performed using 6xHisStCDPK1 [41] as the enzyme source. StCDPK1 is able to phosphorylate the hydrophilic loop of the auxin efflux carrier StPIN4 *in vitro* [49]. However, as observed in Fig 2E, StCDPK1 was unable to phosphorylate StRSG1; the band observed corresponds to the Ca^2+^-dependent autophosphorylation of StCDPK1. These results suggest that StRSG1 could be a specific substrate for StCDPK3.

### *StRSG1* and *StCDPK3* expression analysis

**Fig 3:**
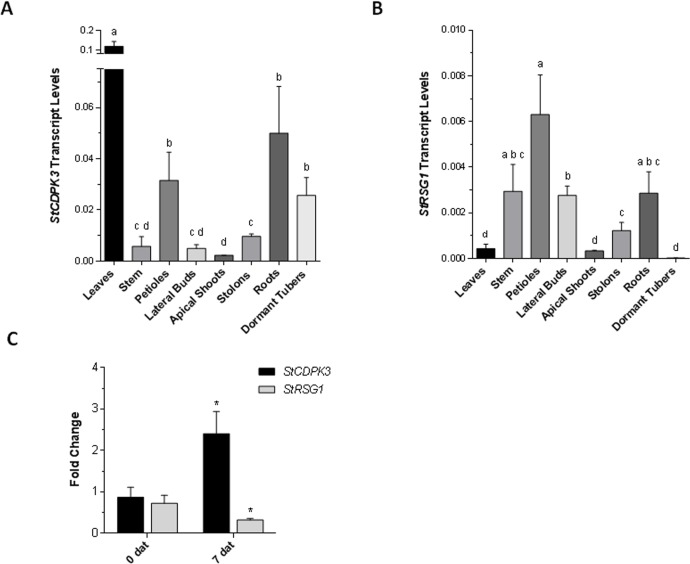
qRT-PCR analysis of *StRSG1* and *StCDPK3*. Expression analysis of *StCDPK3* (A) and *StRSG1* (B) in different plants tissues. Transcript levels were normalized using *Elongation factor-1 α* (*EF-1α*). Means ± SEM of three biological replicates each with three technical replicates were plotted. One-way ANOVA analysis was performed, and Tukey's HSD test was applied. Different letters (a-d) above the bars indicate significant differences in transcript levels between tissues (p<0.05). (C) Analysis of *StCDPK3* and *StRSG1* transcript levels in tubers treated with 5 mg/L GA. Controls were incubated with water; days after treatment (dat) are indicated. *EF-1α* was used for normalization. Means ± SEM of three biological replicates each, with three technical replicates were plotted. The significance of the fold-change in gene expression between the two time points (0 and 7 dat) was evaluated with Student t-test, (*) p<0.05.

RNA-seq data obtained from the PGSC (http://solanaceae.plantbiology.msu.edu/cgi-bin/gbrowse/potato) indicate that *StRSG1* is expressed ubiquitously in the plant; however, a lower expression is observed in stolons, young and mature tubers, shoot apex, petals, and stems. qRT-PCR expression analysis of *StCDPK3* and *StRSG1* transcripts was performed in different tissues from soil-grown plants (Fig 3A and 3B). As observed, *StCDPK3* expression was significantly higher in leaves than in all other tissues, and it was higher in petioles, roots, and tubers compared to stems, lateral buds, apical shoots, and stolons (Fig 3A). Previous results with proSt*CDPK*3:GUS plants showed that, in tubers, St*CDPK*3 expression was restricted to tuber eyes [43]. On the other hand, *StCDPK3* expression in stolons significantly differed from that in apical shoots (Fig 3A). *StRSG1* transcripts were mainly detected in stems, petioles, lateral buds, and roots (Fig 3B). Although low, expression in stolons was statistically significant compared to dormant tubers and apical shoots. According to our data, both transcripts are co-expressed in some tissues and may serve a common pathway.

GA biosynthesis and catabolism play an important role in regulating developmental processes in plants. In potato, high concentrations of GA inhibit tuber development but promote tuber sprouting, thus its concentration must be strictly regulated during these processes [50,51]. We decided to evaluate if *StRSG1* expression was affected by GA. To this end we analyzed the expression of clone STMDI34, encoding a RSG transcriptional activator, in a TIGR 10K microarray assay (GEO series accession number GSE10492) that was performed on tuber sprouts subjected to continuous darkness, continuous light or GA_3_ treatments [52]. STMDI34 expression was similar under dark or light conditions but it was significantly enhanced in sprouts grown under light conditions when GA_3_ was added to the media (x 1.44, p = 0.0024). In addition, non-dormant tubers were incubated for 5 min with water (control) or GA_3_ (treatment) and tubers were processed immediately (time 0) or maintained under long day photoperiod for 7 days after treatment (7dat) to promote sprouting. This time point was chosen based on previous reports [53]. At the moment of harvest (7dat), sprouting was observed in 42% of the GA_3_-treated tubers while only 8% of control tubers sprouted. *StCDPK3* and *StRSG1* expression was analyzed by qRT-PCR in GA-treated and control samples at both time points (0 and 7 dat). As can be observed in Fig 3C, *StCDPK3* expression was significantly induced at 7 dat in response to GA (fold change ~2) compared to 0 dat (*p<0.05). On the other hand, *StRSG1* expression was down-regulated (fold change ~0.4) compared to its expression at 0 dat (*p<0.05). These results indicate that GA modulates *StCDPK3* and *StRSG1* expression.

### StCDPK3 interacts with StABF1 and phosphorylates StABF1 *in vitro*

**Fig 4:**
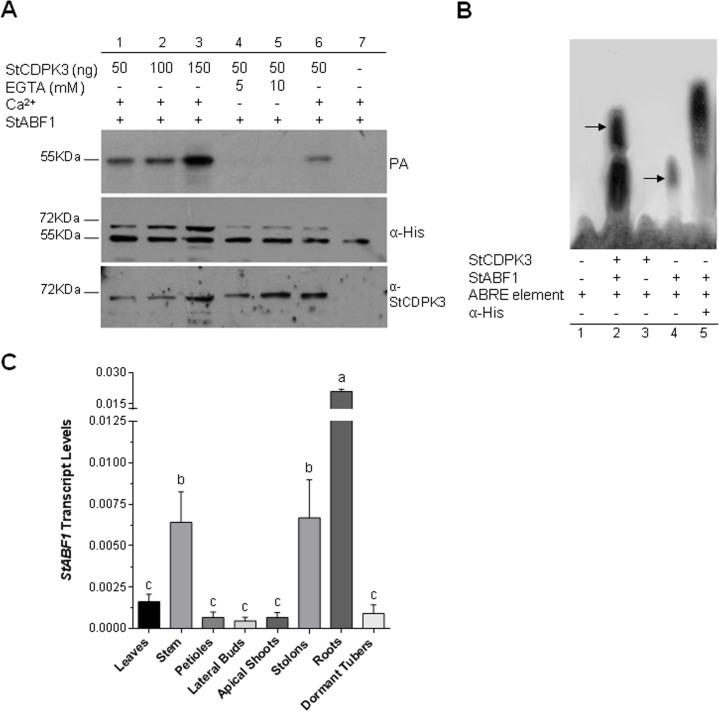
StABF1 is phosphorylated by StCDPK3 and interacts with it. (A) StABF1 phosphorylation assay (PA) was performed using 6xHisStCDPK3 as enzyme source (ng used are indicated in each lane), 6xHisStABF1 (0.5 μg) as phosphate acceptor, and ATPγP^32^ as phosphate donor. Assays were conducted in the presence of 20 μM Ca^2+^ (lanes 1, 2, 3, 6 and 7) or EGTA (lanes 4 and 5). Middle and lower panels: western blot with anti-His (1:5000) and anti-StCDPK3 (NTV, 1:1000) antibodies, respectively. (B) EMSA. The ABRE element was used as a radiolabeled probe. 6xHisStABF1 (1 μg) or 6xHisStCDPK3 (125 ng) were added when indicated. Lane 1: probe alone, lane 2: probe + StABF1 + StCDPK3, lane 3: probe + StCDPK3, lane 4: probe + StABF1, lane 5: probe + StABF1 + anti-His antibody. Shift and super-shift bands are indicated by arrows. (C) qRT-PCR analysis of *StABF1* in different plants tissues. Transcript levels were normalized using *EF-1α*. Means ± SEM of three biological replicates each with three technical replicates were plotted. One-way ANOVA analysis was performed and Tukey's HSD test was applied. Different letters (a-c) above the bars indicate significant differences in transcript levels between tissues (p<0.05).

StABF1 shows all the features of a typical group A bZIP TF of the ABF/AREB family, and binds to the ABRE element *in vitro*. Its expression is induced in response to ABA, drought, salt stress, and cold, and increased during tuber development [54]. StABF1 presents five serine and two threonine residues in an RXXS/T context that could be targeted by kinases and is phosphorylated in response to ABA and salt stress in a calcium-dependent manner. Moreover, StABF1 calcium-dependent phosphorylation is stimulated by tuber-inducing conditions and is inhibited by GA. The potato StCDPK2 isoform phosphorylates StABF1 *in vitro* [54]. Since St*CDPK*3 expression was enhanced by ABA [43], it was worth testing the ability of 6xHisStCDPK3 to phosphorylate StABF1, as an alternative substrate to StRSG1. As depicted in Fig 4A, StCDPK3 phosphorylates StABF1 *in vitro* in a Ca^2+^-dependent way (EGTA addition resulted in no phosphorylation, lanes 4 and 5). The intensity of ABF phosphorylation was stronger with increasing amounts of the kinase (Fig 4A, lanes 1–3) and no signal was detected when StCDPK3 was not included in the mix (lane 7).

In addition, an electrophoretic mobility shift assay (EMSA) was performed with the ABRE element and the recombinant proteins 6xHisStABF1 and 6xHisStCDPK3. As expected, StABF1 was capable of binding to the ABRE element (Fig 4B, lane 4) and the anti-His antibody bound to StABF1 delayed the migration of the StABF1-ABRE complex inducing a super-shift (Fig 4B, lane 5). 6xHisStCDPK3 alone did not bind to the ABRE element (Fig 4B, lane 3); however, when 6xHisStCDPK3 and StABF1 were both present in the reaction, a super-shift was observed confirming an interaction between these proteins (Fig 4B, lane 2). This interaction was independent of StABF1 initial phosphorylation state (data not shown). qRT-PCR expression analysis of *StABF1* transcripts was performed in different tissues from soil-grown plants (Fig 4C). *StABF1* expression was significantly higher in roots, stems, and stolons compared to leaves, petioles, lateral buds, apical shoots, and dormant tubers (Fig 4C).

## Discussion

Protein phosphorylation is a dynamic post-translational modification that permits adaptation to the plant environment. CDPKs are involved in numerous signaling pathways triggered in response to exogenous or endogenous stimuli that result in the activation or inhibition of TFs, which control the expression of downstream genes modulating plant responses [1–3]. In this work, two potato bZIP TFs, StRSG1 and StABF1, were analyzed as downstream targets of StCDPK3.

There are 13 groups (A-L and S) of bZIP TF homologs in angiosperms that represent 34 Possible Groups of Orthologues (PoGOs) [45]. Potato *RSGs*, *RF2a*, *RF2b*, and *VSF-1* genes can be included in the four POGOs identified in group I. Each PoGO represents a group of genes that diverged from an ancestral gene through speciation and duplication [45]. The potato RSG family comprises three closely related members; *StRSG1* and *StRSG2* probably originated from gene duplication. It was reported that Group I contains more genes per PoGO than the average, a fact that suggests that these genes were particularly important for establishing angiosperm-specific physiological or functional characteristics [45].

The mechanisms that regulate GA biosynthesis and catabolism in plants are extremely complex [55]. In Arabidopsis and tobacco, NtRSG contributes to the GA feedback regulation by activating the transcription of genes encoding GA biosynthetic enzymes [19,20]. NtRSG is translocated into the nucleus in response to a reduction in GA levels and GA treatment could reverse this nuclear accumulation [25]. The GA-dependent cytoplasmic migration of RSG requires 14-3-3 binding and Ca^2+^ dependent phosphorylation mediated by NtCDPK1 [16,25]. NtCDPK1 formed a heterotrimer with RSG and 14-3-3 and was postulated as a scaffolding kinase [46]. In this work, we show that 6xHisStCDPK3 was able to phosphorylate NtRSG and StRSG1 *in vitro* in the presence of Ca^2+^. StRSG1 appears to be a specific substrate for StCDPK3 since StCDPK1 was unable to phosphorylate it. StRSG1 contains a serine in position 107 in the same context as Ser-114 in NtRSG [16]. If we infer that in potato, RSG localization is controlled by phosphorylation as in tobacco, Ser-107 should be an interaction site for 14-3-3s. StRSG1 analysis in PredictProtein indicates that Ser-107 is buried when considering solvent accessibility; however, if Ser-107 is replaced by a negatively charged amino acid such as aspartic or glutamic acid, it would be exposed. Based on this *in silico* prediction, we can suggest that when phosphorylated, Ser-107 would be able to interact with a 14-3-3 protein.

Autophosphorylation and substrate phosphorylation are both phosphotransfer reactions catalyzed by StCDPK3; however, while StCDPK3 requires Ca^2+^ for kinase activity, it undergoes autophosphorylation in the absence of Ca^2+^. This is an exception because autophosphorylation is calcium dependent in most CDPKs [56]. Autophosphorylation is a fundamental reaction in eukaryotic protein kinases; activation-loop autophosphorylation (Treo-197 is the site phosphorylated in PKA) is crucial in protein kinases that contain an arginine–aspartic acid (RD) motif within their catalytic loop [57]. StCDPK3 is an RD kinase and when subjected to 2D-gel analysis, multiple autophosphorylation states were revealed [43]. Analysis of StCDPK3 activation loop revealed that it contains Asp in the position equivalent to Treo-197, a feature shared with most CDPKs. However, it presents two potential autophoshorylation sites that are conserved in CDPKs: site I in the activation loop and site II in the CLD [56]. Due to its proximity to the active site, Site I may be important for activation, substrate specificity, or other novel effects on catalysis [56]. It was reported that 14-3-3 binds to the catalytic domain of NtCDPK1 in an autophosphorylation-dependent manner [46]. On the other hand, Site II is located in a loop between EF-hands 1 and 2 and it is unlikely to affect Ca^2+^ binding though it may serve for other function *in vivo* [56]. The paradox is that, in an inactive state, kinases are able to catalyze autophosphorylation [57]. An interesting observation is that StCDPK3 autophosphorylation increased in the presence of StRSG1, suggesting a positive modulation of the kinase by its target. The occurrence of autophosphorylation in different and multiple residues of StCDPK3 could lead to different conformational changes that might alter the binding to specific targets, thus broadening the ability to participate in many signaling pathways.

GAs are negative regulators of tuber induction; environmental stimuli that induce tuberization downregulate GA levels in stolon tips [30,31,58]. *StCDPK3* transcripts were detected in early elongating stolons prior to tuber swelling while *StCDPK1* expression was stronger in swollen stolons [38,49]. According to our qRT-PCR data (Fig 3), both *StCDPK3* and *StRSG1* are present in stolons, thus it is possible to speculate that StCDPK3 could control GA endogenous levels at the onset of tuberization through StRSG1 phosphorylation. StCDPK1 cannot phosphorylate StRSG1 *in vitro* suggesting that both kinases are involved in different signaling cascades. In fact, we recently showed that StCDPK1 is strongly associated with the vascular system and is able to phosphorylate the hydrophilic loop of the auxin transporter StPIN4 *in vitro* [49]. It remains to be confirmed whether StCDPK3 and StRSG1 interact *in vivo*.

We also analyzed the effect of GA on the expression of *StRSG1* and *StCDPK3*. Previously, we observed a decline in *StCDPK3* transcript levels within 6 h of GA_3_ application to *in vitro* grown potato plants [43]. However, when tubers were treated with GA_3_ for 5 min to promote sprouting, an increase in *StCDPK3* transcripts and a decline in *RSG*1 expression were observed (Fig 3C). Moreover, microarray data indicate that GA positively modulates *RSG1* expression in sprouting tubers. Our results show that GA differentially affects the expression of both *StCDPK3* and *StRSG1* in different developmental backgrounds of the potato life cycle. It is interesting that in potato, GA modulates the expression of genes that could regulate its own biosynthesis.

Several reports established a correlation between ABA signaling and CDPK activity or expression. For example, *OsCPK9* transcription was induced by ABA and overexpression of *OsCPK9* resulted in enhanced ABA sensitivity in shoot and root elongation [11]. In *Arabidopsis*, overexpression of *ZmCPK4* enhanced ABA sensitivity in seed germination, seedling growth, and stomatal movement [59]. On the other hand, *Arabidopsis CPK12* is a negative regulator of ABA signaling in seed germination and post-germination growth; *CPK12*-RNAi lines resulted in ABA-hypersensitivity [60]. According to previous results, StABF1 TF mediates the function of ABA during tuberization [54]. Transgenic potato plants that overexpress *ABF4* or *ABF2* from Arabidopsis show increased tuber induction. Under tuber-inducing conditions, *ABF4* overexpression triggers a significant rise in ABA levels and a transcriptional deregulation of GA metabolism genes in stolons [61]. Thus, it could be suggested that ABF TFs function in potato ABA-GA signaling crosstalk during tuberization. *In vitro*, StABF1 is phosphorylated by StCDPK2 [54] and by StCDPK3 (Fig 4). *StCDPK2* and *3* differ in their expression pattern, in their kinetic parameters, and in the Ca^2+^ concentration required for full activation [42,43]. Thus, both kinases could target this TF according to the Ca^2+^ signature received. We also show the formation of a StCDPK3-StABF1-ABRE element interaction complex *in vitro*, reinforcing the hypothesis that these proteins are involved in the same molecular pathway, most likely associated with ABA signaling. Our findings are in accordance with the involvement of CDPKs in signaling pathways triggered by ABA and in the activation of bZIP TFs [60,62–64].

StCDPK3 belongs to a multigene family associated with stress responses and developmental processes; it plays an active role by phosphorylating downstream targets, which in turn activate/repress specific genes. Taken together, our results suggest that StRSG1 is a substrate for StCDPK3, as well as StABF1. Phosphorylation of other ABFs in rice and Arabidopsis was shown to be essential to mediate the ABA signal [65,66]; however, we still do not know how does StABF1 Ca^2+^-dependent phosphorylation affect its activity. It was suggested that the phosphorylation of bZIP TFs in multiple sites can modulate their capability to regulate downstream target genes, allowing the fine-tuning at the DNA-protein interaction level [67]. Recently it was shown that the phosphorylation of AtbZIP63, a bZIP TF from group C, was crucial for its dimerization with different partners and activity *in planta* [68]. Interestingly, bZIP63 is targeted by SnRK1 and AtCPK3 [68]. This TF is an important metabolic regulator of the starvation response and could be a potential point of cross-talk between stress and developmental signals. Considering the effect of *ABF* genes on GA metabolism [61], it is tempting to speculate that StCDPK3 could modulate GA levels in tuberizing stolons by regulating StABF1 and StRSG1. Understanding the complex transcriptional and proteomics dynamics of *S*. *tuberosum* at the onset of tuber development will enlighten the intricate signaling pathways associated to tuberization, and will help to develop new strategies for crop yield improvement in the future.

## Materials and Methods

### Plant material

*S*. *tuberosum* L. cv Desirée wild-type plants were used in this study. *In vitro* plants were micropropagated on a semi solid modified Murashige and Skoog (MS) basal medium supplemented with 2% (w/v) sucrose and 0.7% (w/v) agar. Soil-grown-plants were maintained in the greenhouse, natural light was supplemented 16 h per day by sodium lamps providing 100–300 μmol s^-1^ m^-2,^, the temperature was set at 23°C during day and 19°C in the night. Tissues from 1-month-old-soil-grown- plants were used for RNA isolation.

### Phylogenetic tree construction

Multiple sequence alignments of RSGs full amino acid sequences were made by ClustalW. Potato StRSG1, 2, and 3 (PGSC0003DMP400001527, PGSC0003DMP400050715, and PGSC0003DMP400030704), tomato SlVIP1-like (XP_004237777.1), tobacco NtRSG (BAA97100.1); pepper stress-induced protein 21 (AHI85726.1), *A*. *thaliana* VirE2-interacting protein VIP1 (AAM63070.1), rapeseed TF VIP1-like (NP_001303096.1), soybean and barrel clover (*M*. *truncatula*) bZIP TFs, bZIP131 (NP_001237194.1) and bZIP (XP_013451804.1), rice Os_12g0162500 (Japonica Group) and OsI_37568 (Indica Group), barley predicted protein (BAK03813.1), and sorghum hypothetical protein SORBIDRAFT (XP_002441871.1) were analyzed. A Neighbor-Joining phylogenetic tree was constructed after bootstrap test (n = 500) using the MEGA6 program [69].

### Purification of NtRSG

Dr. Yohsuke Takahashi kindly provided the vector pGEX-4t-NtRSG-GST containing the TF fused to Glutathione S-transferase (GST) for ulterior purification. 100 μl of a starter culture (LB ampicillin 100 mg/L) was used to induce bacterial growth at 37°C to OD 0.4. After adding 1mM IPTG for protein induction, the culture was incubated for 16 h. Cells were collected by centrifugation (15 min at 4,000 x g), and the pellet was resuspended in cold PBS and sonicated. 0.01% Triton X-100 was added and incubated for 30 min, followed by centrifugation (15 min at 10,000 x g) at 4°C. The supernatant containing NtRSG-GST-tagged recombinant protein was incubated for 30 min with 0.5 ml of Glutathione Sepharose*®* 4B Reduced glutathione resin (GSH 4B 50% GE HealthCare). The column was washed three times and eluted afterward.

### Identification, sub-cloning and purification of StRSG1 recombinant protein

*NtRSG* sequence was blasted against the Potato Genome Sequencing Consortium database (http://potato.plantbiology.msu.edu/cgi-bin/gbrowse/potato/). Oligonucleotides StRSG1-FP1 (5´ATGGACCCGAAGTTCACCGG3´) and StRSG1-RP1 (5´ TTAGTTGAAGTTCATGAAGC 3´) were used to amplify the complete coding sequence of *StRSG1* from a cDNA library constructed using Genome Walker Universal Kit. The amplicon obtained was sub-cloned into a pGEX-6p-1 vector (GE, HealthCare) using *BamHI* and *XhoI* cloning sites, and sequenced (GenBank Accession number HQ335343.1). *E. coli* BL21 codon *plus* competent cells were transformed with the construct. The bacterial culture was induced with 1 mM IPTG and culture conditions were adjusted to 25°C for 16 h for optimal protein production. The supernatant containing the recombinant GST-tagged protein was obtained and purified as described above. Western Blot analysis of four eluates was performed with a commercial polyclonal anti-GST antibody (1: 4000, GE HealthCare). Protein content was estimated by [70].

### Purification of 6xHisStCDPK3 and 6xHisStCDPK1

*StCDPK3* and *StCDPK1* coding sequences were cloned in pDEST 17 Vector (Invitrogen) to obtain the His-tagged recombinant proteins [41,43]. 6xHisStCDPK3 and 6xHisStCDPK1 proteins were efficiently produced after overnight induction of *E*. *coli* BL21 codon plus cells with 1 mM IPTG at 25°C. The tagged proteins were purified by affinity chromatography, using a nickel agarose column (Ni-NTA agarose, QIAGEN) according to manufacturer’s procedures, as already described [43].

### Phosphorylation assays

CDPK activity was assayed according to [42] in a reaction mixture containing 25 mM Tris–HCl, pH 7.5, 10 mM MgCl_2_, 20 μM CaCl_2_, 10 mM β-mercaptoethanol, 10 μM [γ-^32^P]-ATP (specific activity 2,331 Bq pmol^-1^, New England Nuclear) and NtRSG-GST (0.6 to 6 μg), StRSG1-GST (0.5 to 2 μg), or 6xHisStABF1 (0.5 μg; GenBank accession number HM988989.1) as substrates. Purified 6xHisStCDPK3 or 6xHisStCDPK1 (50–150 ng) were used as kinase source. Reactions were incubated at 30°C for 5 minutes, and samples were resolved by 10% SDS-polyacrylamide gel electrophoresis and transferred to nitrocellulose membranes. Autoradiographs were obtained and images were scanned. ImageJ was used to quantify the relative area of the phosphorylation signal. Western blots were performed using a polyclonal antibody against a specific sequence (KYTQQDANGYRAGRC) of StCDPK3 NTV domain (1:1000, produced by Genescript), or antibodies anti-His (1:5000; GE Healthcare, 27-4710-01) or anti-GST (1:4000; GE Healthcare 27-4577-01) as indicated.

### Analysis of *CDPK*3 and TF transcripts levels

*StCDPK3*, *StRSG1*, and *StABF1* transcript levels were determined by qRT-PCR. Different tissues were harvested from 1-month-old-soil-grown plants, and RNA was isolated by TRIzol (Invitrogen) following the manufacturer’s instructions. RNA was quantified, using a Nanodrop 1000 (Thermo Scientific), and checked by 1.4% agarose gels. Total RNA (1 μg) was treated with DNase (Promega) and used for cDNA synthesis using oligodT oligonucleotides. All qPCR reactions were performed on Applied Biosystems™ 7500 Real-Time PCR Systems using FastStart Universal SYBR Green Master mixture (Roche). For expression analysis, the following oligonucleotides were used: StCDPK3-FP 5´AGCCAGAAGGGCCATATCA3´, StCDPK3-RP 5´GTCCAGGCTGCACAGTAACA3´, StRSG1-FP 5´ACCTTGACCAGATGCCGG3´, StRSG1-RP 5´CGGCTGCATGTGGGTATC3´, StABF1-FP 5´AGTCAGCTGCTAGATCAAGAGC3´, StABF1-RP 5´AAGTCTGTAATATGGCTAATCCAC3´. *StCDPK3* and *StRSG1* reactions were carried out at 95˚C for 10 min, followed by 40 cycles of 95˚C for 15 s, and 60˚C for 1 min. *StABF1* reactions were performed at 95˚C for 10 min, followed by 40 cycles of 95˚C for 15 s, 50°C for 20 s and 60˚C for 1 min. *Elongation factor-1 α* was used for normalization using EF-1α-FP (5´TGAGGCAAACTGTTGCTGTC 3´) and EF-1α-RP (5´TGGAAACACCAGCATCACAC3´) primers [71]. PCR specificity was checked by melting curve analysis and data was analyzed using the 2^-(Ct gene of interest -Ct EF-1α)^ method [72]. Subsequently, one-way ANOVA analysis was performed and Tukey's HSD test was applied. Means ± SEM of three biological replicates each, with three technical replicates are plotted. Graphs were plotted using Graphpad Prism (6.07 version) software.

To analyze the effect of GA on *StCDPK3* and *StRSG1* transcript levels, *in vitro* 2-months-old post-harvest tubers were treated during 10 min with GA_3_ (5 mg/L) or water (control). One batch was immediately frozen in liquid nitrogen (0 dat), and the other batch was placed in MS 2% sucrose medium and incubated in long day photoperiod (16:8 h) for 7 days (7 dat). Whole tubers were collected to isolate total RNA, and cDNA was obtained. Real Time qPCR was performed as mentioned before. Fold-changes in *StCDPK3* and *StRSG1* expression of GA-treated versus control samples were calculated using the 2^-∆∆Ct^ method [73]. The significance of the fold-change in gene expression between the two time points (0 and 7 dat) was evaluated by the Student t-test (p<0.05). Means ± SEM of three biological replicates each, with three technical replicates were plotted.

### Electrophoretic Mobility Shift Assay (EMSA)

Recombinant 6XHisStABF1 (1 μg), previously phosphorylated or not, was incubated with 6xHisStCDPK3 (125 ng) or with anti-His antibody. ABRE oligonucleotides sense and antisense (aattccGGACACGTGGCGtaagct) were used as probes. 100 pmol of oligonucleotides (probes) were annealed by heating at 100°C during 5 min, followed by slow cooling to facilitate interaction. The ABRE probe was labeled using dCTP[α^32^P] and T4 polynucleotide kinase (*New England Biolabs*, Ipswich, MA). For interaction assays, 6xHisStABF1 was incubated during 15 min at 4°C with 1.5 μg of poly (dl-dC), in 30 μl buffer containing: 10 mM Tris-HCl, pH 7.5, 2 mM MgCl_2_, 100 mM NaCl, 1 mM EDTA, 4% (v/v) glycerol, and 1 mM DTT. Then, 1.5 ng of ABRE probe was added to the reaction and incubated during 15 min at room temperature. Reactions were solved in 6% acrylamide gels in buffer TBE 0.5x (45 mM Tris Base, 45 mM boric acid, 1 mM EDTA). Gels were exposed during 1 h to a cassette with an amplifying signal screen, and signal was recorded using a Storm 820 PhosphorImager (*Amersham Pharmacia Biotech*)

## Supporting Information

S1 Fig **Genomic context of *StRSG1* (A) and *StRSG2* (B) genes in chromosome 4.** The chromosome region between 59 and 59.5 Mbp is depicted in the upper panel; the inverted repeats (stripped arrows) are separated by a 273 kbp spacer region. In A and B scale bars indicate the position of the genes according to PGSC (http://solanaceae.plantbiology.msu.edu/cgi-bin/gbrowse/potato/). Grey arrows represent *StRSG1* and *StRSG2* and the corresponding upstream and downstream genes. Gene orientation is indicated. Striped arrows span the regions of the duplicated inverted repeats. *CYP450*, cytochrome P450; *SDC*, serine decarboxylase.
(TIF)

S1 Table StRSG1 blast hits in the PGSC database. StRSG1 complete protein sequence (upper chart) or its bZIP domain (lower chart) were blasted (BLASTP 2.2.26) against the PGSC DM v3.4 pep.fasta database (http://solanaceae.plantbiology.msu.edu/blast.shtml.). Q coverage and identities are shown.
(PDF)

S2 Table Localization of *StRSG1*, *2* and *3* genes in the potato genome. PGSC DMG, DMT, and DMP accessions for the potato RSG genes are indicated. Chromosome position, exon and intron length, and gene length are indicated in bp. The MW of the protein is indicated.
(DOCX)
