# Supplementary figures and images for: StCDPK3 Phosphorylates In Vitro Two Transcription Factors Involved in GA and ABA Signaling in Potato: StRSG1 and StABF1
Source: PLoS One. 2016 Dec 1;11(12):e0167389. doi: 10.1371/journal.pone.0167389 (PMC5131985; doi:10.1371/journal.pone.0167389)

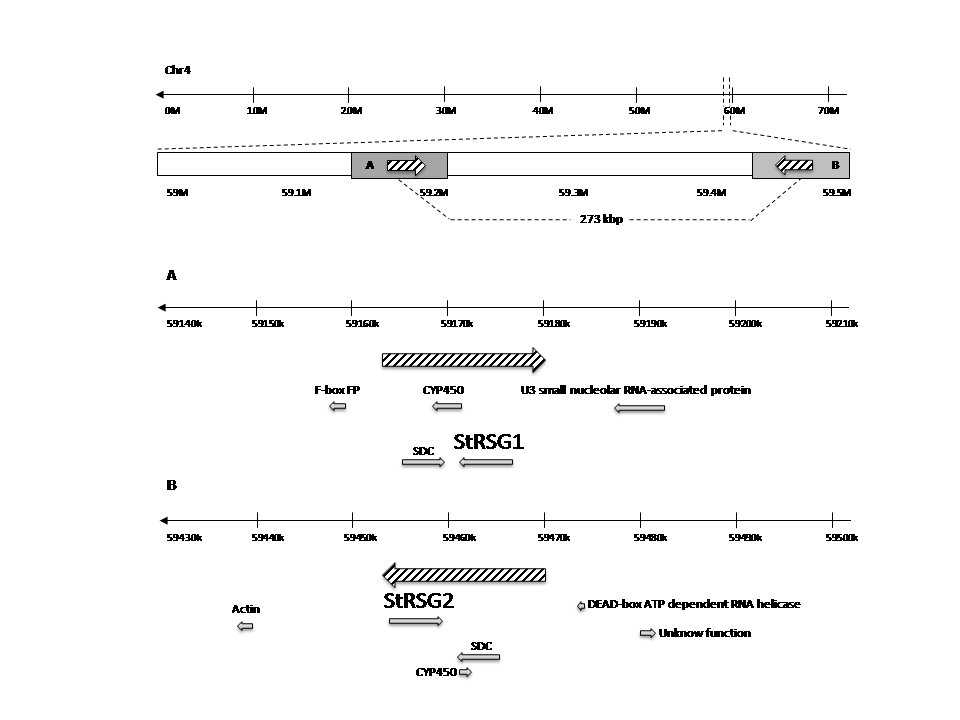

Supplement: S1 Fig — Genomic context of StRSG1 (A) and StRSG2 (B) genes in chromosome 4. The chromosome region between 59 and 59.5 Mbp is depicted in the upper panel; the inverted repeats (stripped arrows) are separated by a 273 kbp spacer region. In A and B scale bars indicate the position of the genes according to PGSC (http://solanaceae.plantbiology.msu.edu/cgi-bin/gbrowse/potato/). Grey arrows represent StRSG1 and StRSG2 and the corresponding upstream and downstream genes. Gene orientation is indicated. Striped arrows span the regions of the duplicated inverted repeats. CYP450, cytochrome P450; SDC, serine decarboxylase. (TIF) [file pone.0167389.s001.tif]
