# Supplementary material for: StCDPK3 Phosphorylates In Vitro Two Transcription Factors Involved in GA and ABA Signaling in Potato: StRSG1 and StABF1
Source: PLoS One. 2016 Dec 1;11(12):e0167389. doi: 10.1371/journal.pone.0167389 (PMC5131985; doi:10.1371/journal.pone.0167389)
Supplement: S1 Table — StRSG1 complete protein sequence (upper chart) or its bZIP domain (lower chart) were blasted (BLASTP 2.2.26) against the PGSC DM v3.4 pep.fasta database (http://solanaceae.plantbiology.msu.edu/blast.shtml.). Q coverage and identities are shown. (PDF) [file pone.0167389.s002.pdf]

S1 Table. StRSG1 blast hits in the PGSC database

Query: StRSG1 complete coding sequence

Accession	Description	Hit Score	E value	Num HSPs	Top Query Cov (%)	Top Id (%)
PGSC0003DMP400001527	BZIP transcriptional activator RSG	1792	0.0	1	100.00	100.00
PGSC0003DMP400050715	BZIP transcriptional activator RSG	1317	0.0	1	77.35	96.96
PGSC0003DMP400030704	BZIP transcriptional activator	1194	1,00E-165	1	99.12	74.27
PGSC0003DMP400050716	BZIP transcriptional activator RSG	897	1,00E-122	1	54.41	95.68
PGSC0003DMP400029125	Transcription factor RF2b	492	2,00E-59	1	75.00	49.62
PGSC0003DMP400047996	Transcription factor RF2b	471	5,00E-56	1	92.06	43.08
PGSC0003DMP400002986	DNA binding protein	458	5,00E-52	1	53.24	56.35
PGSC0003DMP400006634	Transcription factor VSF-1	409	7,00E-46	1	55.00	51.06
PGSC0003DMP400021996	Transcription factor RF2a	231	9,00E-22	1	28.24	47.92
PGSC0003DMP400016592	Transcription factor RF2a	226	6,00E-21	1	27.94	49.47

Query: StRSG1 b-ZIP domain

Accession	Description	Hit Score	E value	Num HSPs	Top Query Cov	Top Id
PGSC0003DMP400001527	BZIP transcriptional activator RSG	461	2,00E-58	1	100.00	100.00
PGSC0003DMP400050715	BZIP transcriptional activator RSG	440	4,00E-56	1	100.00	96.74
PGSC0003DMP400030704	BZIP transcriptional activator	409	9,00E-51	1	100.00	89.13
PGSC0003DMP400050716	BZIP transcriptional activator RSG	388	3,00E-49	1	88.04	95.06
PGSC0003DMP400029125	Transcription factor RF2b	358	4,00E-43	1	100.00	78.26
PGSC0003DMP400002986	DNA binding protein	367	1,00E-42	1	100.00	78.26
PGSC0003DMP400047996	Transcription factor RF2b	346	5,00E-41	1	100.00	75.00
PGSC0003DMP400006634	Transcription factor VSF-1	303	3,00E-34	1	100	67.39
PGSC0003DMP400016592	Transcription factor RF2a	209	3,00E-21	1	100.00	51.09
PGSC0003DMP400021996	Transcription factor RF2a	207	4,00E-21	1	100.00	50.00

StRSG1 complete protein sequence (upper chart) or its bzip domain (lower chart) were blasted (BLASTP 2.2.26) against the PGSC DM v3.4 pep.fasta database (http://solanaceae.plantbiology.msu.edu/blast.shtml.). Q coverage and identities are shown.
