# Supplementary material for: StCDPK3 Phosphorylates In Vitro Two Transcription Factors Involved in GA and ABA Signaling in Potato: StRSG1 and StABF1
Source: PLoS One. 2016 Dec 1;11(12):e0167389. doi: 10.1371/journal.pone.0167389 (PMC5131985; doi:10.1371/journal.pone.0167389)
Supplement: S2 Table — PGSC DMG, DMT, and DMP accessions for the potato RSG genes are indicated. Chromosome position, exon and intron length, and gene length are indicated in bp. The MW of the protein is indicated. (DOCX) [file pone.0167389.s003.docx]

**S2 Table. Localization of StRSG1, 2 and 3 genes in the potato genome**

|  | PGSC0003 Accessions | | |  |  |  | from ATG to STOP codon (bp) | | | | | | |  |
| --- | --- | --- | --- | --- | --- | --- | --- | --- | --- | --- | --- | --- | --- | --- |
| **Description** | **DMG** | **DMT** | **DMP** | **chr** | **Localization** | **Genomic sequence bp** | **Exon 1** | **Exon** | **Exon 3** | **Exon 4** | **Intron 1** | **Intron 2** | **Intron 3** | **MW (kDa)** |
| *StRSG1* | 400000799 | 400002100 | 400001527 | 4 | 59168900…59175200 | 6300 | 499 | 132 | 84 | 243 | 1172 | 3568 | 82 | 37 |
| *StRSG2* | 400029118 | 400074878 | 400050715 | 4 | 59452426...59459127 | 6701 | 327 | 132 | 84 | 237 | 1188 | 3638 | 83 | 29 |
|  |  | 400074879 | 400050716 |  |  |  | 327 | 132 | 84 | 0 | 1188 | 3638 |  |  |
| *StRSG3* | 400017577 | 400045314 | 400030704 | 6 | 43451328..43458453 | 7125 | 525 | 132 | 84 | 240 | 1176 | 3028 | 1588 | 36 |

The corresponding PGSC accessions are indicated. Chromosome position, gene length and the length of exons and introns are indicated in bp. Chr: chromosome
